# Advancing cancer therapy: new frontiers in targeting DNA damage response

**DOI:** 10.3389/fphar.2024.1474337

**Published:** 2024-09-20

**Authors:** Jiekun Qian, Guoliang Liao, Maohui Chen, Ren-Wang Peng, Xin Yan, Jianting Du, Renjie Huang, Maojie Pan, Yuxing Lin, Xian Gong, Guobing Xu, Bin Zheng, Chun Chen, Zhang Yang

**Affiliations:** ^1^ Department of Thoracic Surgery, Fujian Medical University Union Hospital, Fuzhou, China; ^2^ Fujian Key Laboratory of Cardiothoracic Surgery, Fujian Medical University, Fuzhou, China; ^3^ Clinical Research Center for Thoracic Tumors of Fujian Province, Fuzhou, China; ^4^ Division of General Thoracic Surgery, Department of BioMedical Research (DBMR), Inselspital, Bern University Hospital, University of Bern, Bern, Switzerland; ^5^ Department of Cardiac Medical Center Nursing, Fujian Medical University Union Hospital, Fuzhou, China

**Keywords:** genomic instability, DNA damage response, vulnerability, synthetic lethality, resistance

## Abstract

Genomic instability is a core characteristic of cancer, often stemming from defects in DNA damage response (DDR) or increased replication stress. DDR defects can lead to significant genetic alterations, including changes in gene copy numbers, gene rearrangements, and mutations, which accumulate over time and drive the clonal evolution of cancer cells. However, these vulnerabilities also present opportunities for targeted therapies that exploit DDR deficiencies, potentially improving treatment efficacy and patient outcomes. The development of PARP inhibitors like Olaparib has significantly improved the treatment of cancers with DDR defects (e.g., BRCA1 or BRCA2 mutations) based on synthetic lethality. This achievement has spurred further research into identifying additional therapeutic targets within the DDR pathway. Recent progress includes the development of inhibitors targeting other key DDR components such as DNA-PK, ATM, ATR, Chk1, Chk2, and Wee1 kinases. Current research is focused on optimizing these therapies by developing predictive biomarkers for treatment response, analyzing mechanisms of resistance (both intrinsic and acquired), and exploring the potential for combining DDR-targeted therapies with chemotherapy, radiotherapy, and immunotherapy. This article provides an overview of the latest advancements in targeted anti-tumor therapies based on DDR and their implications for future cancer treatment strategies.

## 1 Introduction

**FIGURE 1:**
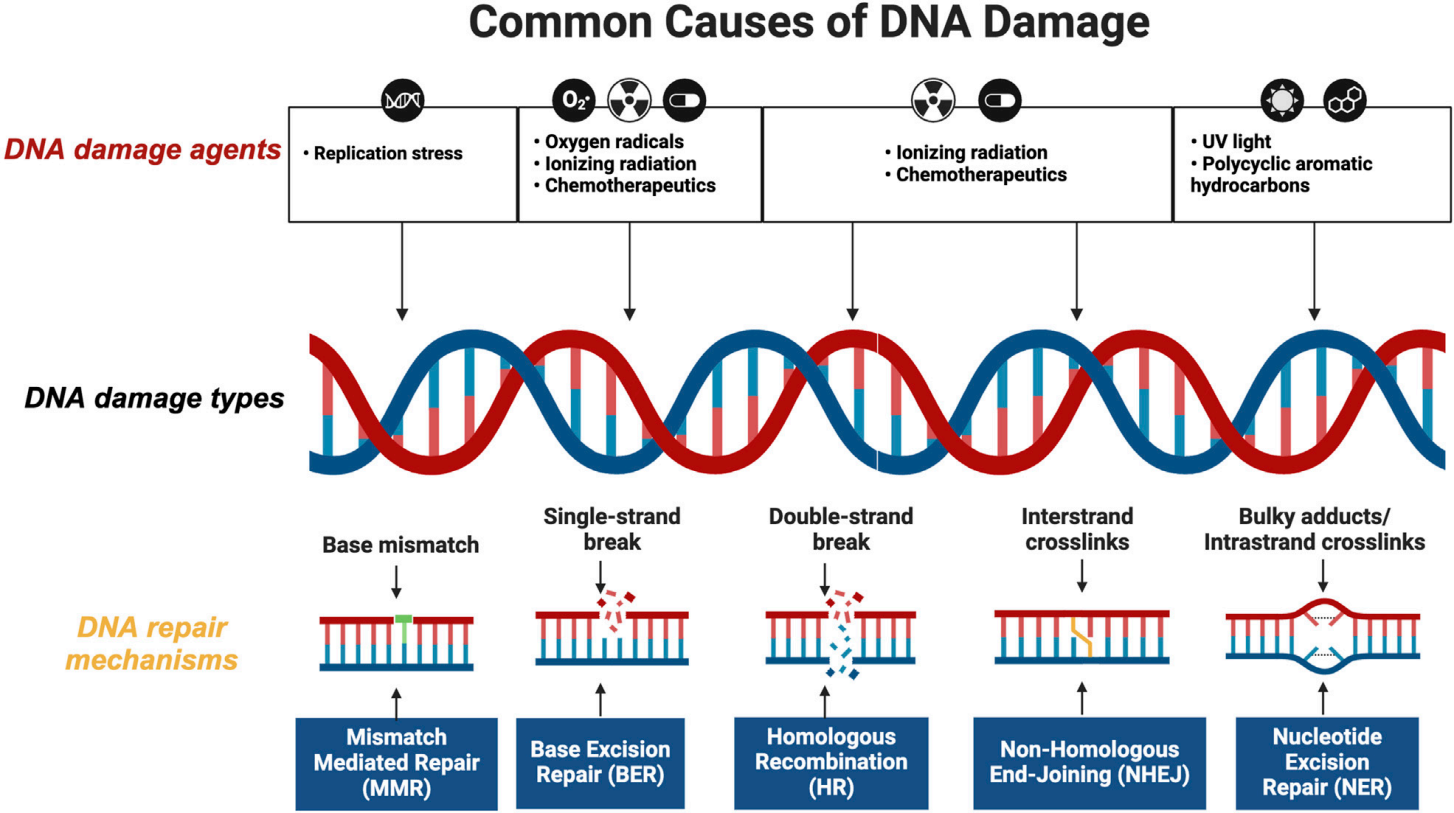
Overview of DNA Damage Response (DDR) Mechanisms Ensuring Cellular Viability and Genome Integrity. This figure illustrates the sophisticated network of DDR mechanisms that cells employ to maintain genome integrity. Central to the DDR are five key pathways: Non-Homologous End Joining (NHEJ), Homologous Recombination (HR), Mismatch Repair (MMR), Nucleotide Excision Repair (NER), and Base Excision Repair (BER). Each pathway is depicted with its specific role and interaction within the cellular environment to repair various types of DNA damage (created with BioRender.com, accessed on 25 August 2024).

DNA damage response (DDR) is crucial for maintaining genome stability (Brandsma et al., 2017). Research shows that cells are constantly exposed to DNA damage from various sources, including UV radiation, ionizing radiation, chemical exposure, replication errors, cellular metabolism, and oxidative stress. These factors can cause either DNA single-strand breaks (SSBs) or DNA double-strand breaks (DSBs) (Malaquin et al., 2015; O’Connor, 2015). Cells utilize sophisticated DDR mechanisms to ensure cellular viability and genome integrity, such as non-homologous end joining (NHEJ), homologous recombination (HR), mismatch repair (MMR), nucleotide excision repair (NER), base excision repair (BER) (Figure 1). These systems are essential for DNA damage recognition, cell cycle arrest, DNA damage repair, and apoptosis initiation in cells with irreparable damage (Basu et al., 2012; Nickoloff et al., 2017).

**FIGURE 2:**
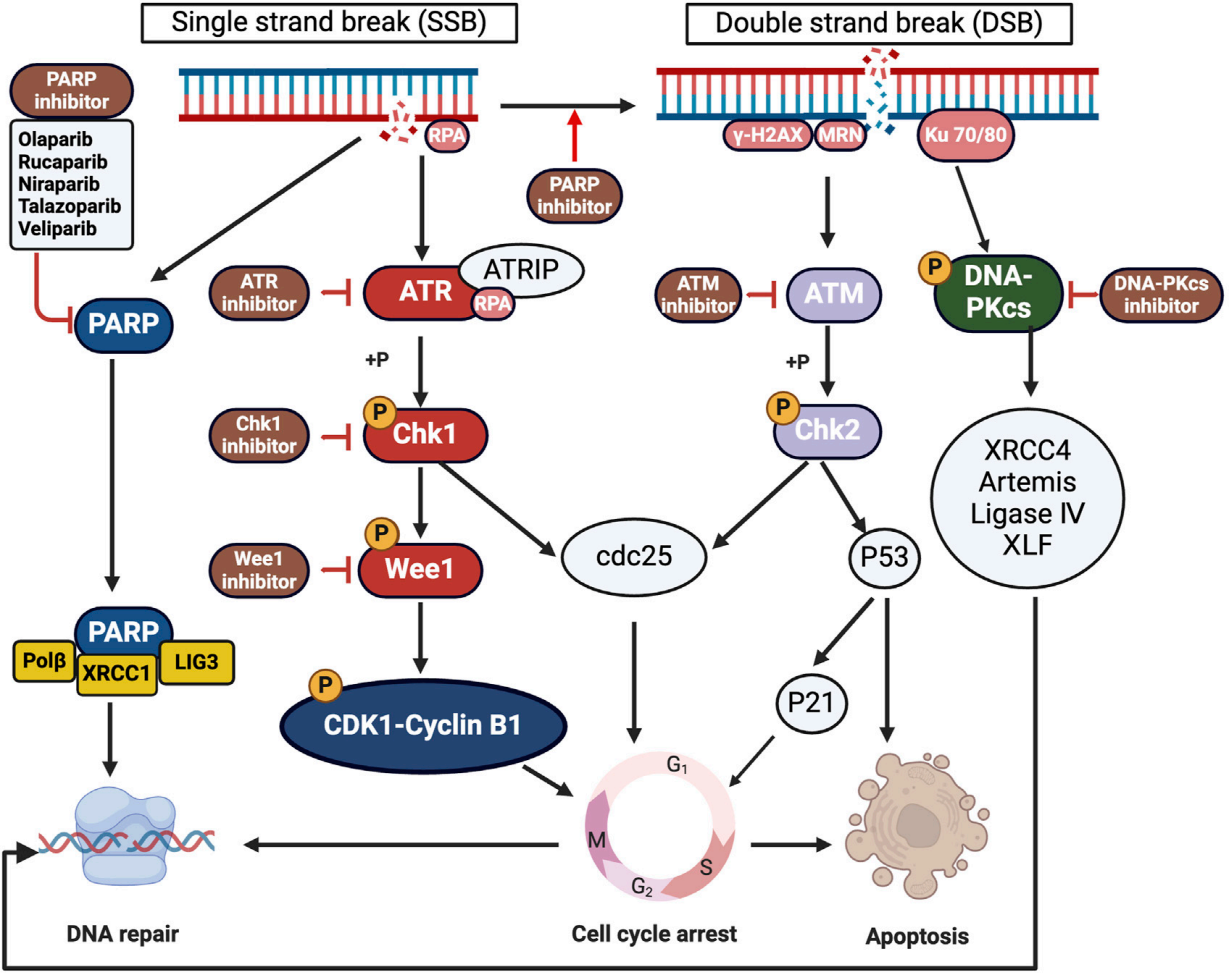
Targeting Overexpressed DNA Repair Proteins in Cancer Therapy. This figure details the critical proteins in cancer cells central to DNA repair mechanisms, contributing to treatment resistance. Highlighted proteins include PARP, DNA-PKcs, ATM, ATR, Wee1 and Chk1/2, which enhance DNA repair and contribute to treatment resistance. The diagram also shows inhibitors developed to disrupt these pathways, depicting how each inhibitor interacts with its target protein to increase cancer cell sensitivity to treatments and potentially overcome resistance (created with BioRender.com, accessed on 25 August 2024).

Cancer cells often exhibit elevated levels of DNA damage repair proteins, allowing them to survive and proliferate despite DNA damage induced by chemotherapy or radiotherapy. Essential proteins frequently overexpressed in cancer cells include PARP, DNA-PKcs, BRCA1/2, ATM, ATR, and Chk1/2 (Kim et al., 2020; Okabe et al., 2023; Yang et al., 2020; Jin et al., 2022; Savva et al., 2019; Obata et al., 2023; Dilmac and Ozpolat, 2023; Tang et al., 2024; Gralewska et al., 2020). This overexpression facilitates DNA damage repair and contributes to treatment resistance. To counter this, scientists have developed inhibitors targeting these proteins to disrupt DNA repair processes in cancer cells (Figure 2). This approach effectively enhances the impact of therapy-induced DNA damage, thereby increasing the likelihood of inducing apoptosis in cancer cells and potentially improving treatment outcomes.

Conversely, defects in DDR pathways can lead to mutations and increased genomic instability, driving cancer initiation and progression. Cancer cells often have rapid division rates and are more vulnerable to specific DDR inhibitors like ATR and DNA-PK inhibitors. Exploiting this vulnerability allows targeted therapy to differentiate between normal cells with intact DDR and cancer cells with DDR defects (Pilié et al., 2019; Basourakos et al., 2017; McPherson and Korzhnev, 2021). Based on synthetic lethality, this innovative approach selectively eliminates cancer cells while sparing normal cells (Minchom et al., 2018), exemplified by the efficacy of ATR inhibitors in ATM-deficient cancers and Wee1 inhibitors in p53-mutant cancers (O’Neil et al., 2017). Synthetic lethality occurs when cell death is induced by simultaneous defects in two or more related genes, whereas a single defect alone might not compromise cell survival (Huang et al., 2020; Mullard, 2017). Advances in gene editing technologies such as RNAi and CRISPR have enabled large-scale screening of synthetic lethal targets, leading to new therapeutic discoveries.

PARP inhibitors are a notable application of synthetic lethality, effectively targeting tumors with DDR defects like BRCA1/2 gene mutations (Bryant et al., 2005; Farmer et al., 2005). Several PARP inhibitors have received FDA approval for cancer treatment, including Talazoparib, Rucaparib, Niraparib, and Olaparib (Min and Im, 2020; Pascal, 2018; Slade, 2020). Synthetic lethality-based strategies offer several advantages, such as overcoming resistance to traditional therapies and producing synergistic anticancer effects when combined with radiotherapy or chemotherapy (Lord and Ashworth, 2017; Hu and Guo, 2020; Tang et al., 2020). The discovery of numerous therapeutically relevant molecules has spurred increased interest in synthetic lethality-based therapies (Hengel et al., 2017). Many novel DDR-targeting molecules are undergoing clinical trials with promising results (Table 1) (Fok et al., 2019; Ricciuti et al., 2020; Sheng et al., 2020; Wengner et al., 2020). This paper summarizes the biological characteristics and limitations of various DDR inhibitors and reviews recent advancements in clinical research.

**TABLE 1:** DDR inhibitors in clinical trial.

Pathway	Target	Compound	Stage	Disease	Clinical trial dentifier and status
NHEJ					
	DNA-PKcs	AZD7648	Phase Ⅰ/Ⅱ	Advanced malignancy Adult soft tissue sarcoma	NCT03907969 (COMPLETED) NCT05116254 (RECRUITING)
		M9831	Phase Ⅰ	Advanced solid tumor	NCT02644278 (COMPLETED)
		M3814	Phase Ⅰ	Glioblastoma Gliosarcoma Ovarian Cancer	NCT04555577 (RECRUITING) NCT04092270 (RECRUITING)
		CC-115	Phase Ⅰ	Prostate cancer Advanced malignancy	NCT02833883 (PMID: 37980367) NCT01353625 (PMID: 31853198)
			Phase Ⅱ	Glioblastoma	NCT02977780 (PMID: 37722087)
BER					
	PARP	E7016	Phase Ⅱ	Melanoma	NCT01605162 (TERMINATED)
		Niraparib	Phase Ⅲ	Ovarian cancer Breast cancer	NCT01847274 (PMID: 36970052) NCT01905592 (TERMINATED)
		Olaparib	Phase Ⅰ	Lung cancer Carcinoma of the oesophagus Head and neck cancer Breast carcinoma	NCT02511795 (COMPLETED) NCT01460888 (UNKNOWN STATUS) NCT01562210 (PMID: 31500595) NCT01758731 (COMPLETED) NCT02308072 (ACTIVE, NOT RECRUITING) NCT02227082 (COMPLETED) NCT02229656 (PMID: 31500595)
			Phase Ⅲ	Ovarian cancer Fallopian tube cancer Breast cancer Gastric cancer Pancreatic cancer Primary peritoneal cancer	NCT01844986 (PMID: 36082969) NCT01874353 (PMID: 35772665) NCT01924533 (PMID: 29103871) NCT02000622 (PMID: 32472001) NCT02032823 (PMID: 38301187) NCT02184195 (PMID: 38687918) NCT02282020 (PMID: 32073956) NCT02392676 (WITHDRAWN) NCT02446600 (PMID: 35290101) NCT02477644 (PMID: 31851799) NCT02502266 (ACTIVE, NOT RECRUITING)
			Phase Ⅳ	Ovarian cancer	NCT02476968 (PMID: 37030280)
		Rucaparib	Phase Ⅱ	Prostate cancer	NCT03413995 (PMID: 38885246)
			Phase Ⅲ	Ovarian carcinoma	NCT01968213 (PMID: 37262961)
		Talazoparib	Phase Ⅲ	Prostate cancer Breast cancer	NCT03395197 (PMID: 37285865) NCT01945775 (PMID: 38886516)
		Veliparib	Phase Ⅰ/Ⅱ	Advanced solid malignancy with peritoneal carcinomatosis Epithelial ovarian cancer Fallopian cancer Primary peritoneal cancer Breast cancer Pancreatic cancer Non-small cell lung cancer Diffuse pontine gliomas	NCT01264432 (COMPLETED) NCT01477489 (PMID: 29558281) NCT01514201 (PMID: 32009149) NCT01618357 (COMPLETED) NCT01908478 (COMPLETED) NCT02412371 (TERMINATED)
			Phase Ⅲ	Breast cancer Non-Small cell lung cancer Glioblastoma Gliosarcoma Ovarian cancer	NCT02032277 (PMID: 33599688) NCT02106546 (PMID: 34436928) NCT02152982 (PMID: 26615020) NCT02163694 (PMID: 32861273) NCT02264990 (PMID: 35331641) NCT02470585 (PMID: 34930617)
HR					
	ATR	AZD6738	Phase Ⅰ	Refractory cancer	NCT02630199 (COMPLETED)
			Phase Ⅱ	Gastric adenocarcinoma Malignant melanoma	NCT03780608 (UNKNOWN STATUS)
		M6620	Phase Ⅰ	Oesophageal adenocarcinoma Solid tumor Squamous cell carcinoma	NCT03641547 (PMID: 38129525) NCT02487095 (PMID: 29252124)
		BAY-1895344	Phase Ⅰ	Solid tumor Ovarian cancer Non-Hodgkin’s lymphoma	NCT04267939 (TERMINATED) NCT03188965 (COMPLETED)
		M4344	Phase Ⅰ	Ovarian cancer Solid tumor	NCT04149145 (WITHDRAWN) NCT02278250 (COMPLETED)
			Phase Ⅰ/Ⅱ	Advanced solid tumor Breast cancer	NCT04655183 (WITHDRAWN)
		M1774	Phase Ⅰ	Endometrial carcinoma Ovarian carcinoma Solid tumor	NCT06308263 (RECRUITING) NCT05950464 (RECRUITING) NCT05396833 (RECRUITING) NCT05687136 (RECRUITING)
			Phase Ⅱ	Merkel cell carcinoma Refractory prostate carcinoma	NCT05947500 (RECRUITING) NCT05828082 (RECRUITING)
			Phase Ⅰ/Ⅱ	Advanced microsatellite stable colorectal carcinoma Hematopoietic and lymphatic system neoplasm Non-small cell lung cancer	NCT05691491 (RECRUITING) NCT05882734 (RECRUITING)
	ATM	AZD1390	Phase Ⅰ	Brain cancer Glioblastoma Glioblastoma multiforme Glioma Adult soft tissue sarcoma Non small cell lung cancer Healthy volunteer male subjects Solid tumor	NCT03423628 (RECRUITING) NCT05182905 (RECRUITING) NCT05116254 (RECRUITING) NCT05678010 (RECRUITING) NCT04550104 (RECRUITING) NCT03215381 (COMPLETED)
		AZD0156	Phase Ⅰ	Solid tumor	NCT02588105 (COMPLETED)
Cell Cycle Checkpoint					
	Chk1	GDC-0575	Phase Ⅰ	Lymphoma Solid tumor	NCT01564251 (PMID: 29788155)
		LY-2606368	Phase Ⅱ	Ovarian cancer	NCT03414047 (PMID: 36192237)
		SRA737	Phase Ⅰ/Ⅱ	Solid tumor Non-Hodgkin’s lymphoma	NCT02797964 (PMID: 37120671)
		MK-8776	Phase Ⅰ	Hodgkin disease Non-Hodgkin’s lymphoma Leukemia Adavance solid tumor	NCT00779584 (PMID: 25605849) NCT00907517 (TERMINATED) NCT01521299 (WITHDRAWN)
			Phase Ⅱ	Leukemia	NCT01870596 (PMID: 28957699)
	Wee1	Debio 0123	Phase Ⅰ	Solid tumor	NCT03968653 (RECRUITING)
		SY-4835	Phase Ⅰ	Advanced Solid tumor	NCT05291182 (RECRUITING)
		IMP7068	Phase Ⅰ	Advanced Solid tumor	NCT04768868 (RECRUITING)
		AZD1775	Phase Ⅰ	Solid tumors Ovarian cancer	NCT02610075 (WITHDRAWN)
			Phase Ⅱ	Ovarian cancer Fallopian tube cancer Peritoneal cancer Pancreatic cancer Acute myeloid leukemia	NCT02272790 (PMID: 34645648) NCT01357161 (PMID: 32611648) NCT02037230 (PMID: 31398082) NCT02791919 (WITHDRAWN)
		ZN-c3	Phase Ⅰ	Fallopian tube carcinoma Ovarian carcinoma Peritoneal carcinoma Breast cancer Lung cancer Pancreatic cancer Solid tumor	NCT05368506 (WITHDRAWN) NCT05431582 (WITHDRAWN) NCT04158336 (RECRUITING) NCT04516447 (RECRUITING)
			Phase Ⅱ	Pancreatic cancer	NCT06015659 (RECRUITING)
			Phase Ⅰ/Ⅱ	Acute myeloid leukemia Metastatic colorectal cancer Breast cancer Uterine serous carcinoma Osteosarcoma	NCT05682170 (RECRUITING) NCT05743036 (RECRUITING) NCT06351332 (RECRUITING) NCT04814108 (ACTIVE, NOT RECRUITING) NCT04833582 (ACTIVE, NOT RECRUITING)

## 2 Historical development of DDR-targeted therapies in cancer

In the 1970s and 1980s, groundbreaking research on DNA repair mechanisms laid the foundation for understanding how cells detect and repair DNA damage. During this time, key pathways like NER, BER, and MMR were identified and characterized. These discoveries paved the way for significant advancements in the late 1980s–1990s, such as the discovery of the ATM gene and its crucial role in the DNA damage response. Additionally, the discovery of BRCA1 and BRCA2 genes highlighted their roles in HR, linking mutations in these genes to increased risks of breast and ovarian cancers (Sancar, 1995). By the early 2000s, the focus of research shifted to targeting specific DDR proteins, leading to the development of synthetic lethality strategies. This approach was particularly effective in BRCA-deficient cancers, exemplified using PARP inhibitors (Farmer et al., 2005; Fong et al., 2009). During this period, substantial advancements were achieved, particularly with the introduction of Olaparib, the first PARP inhibitor, into clinical trials. Olaparib exhibited efficacy in targeting cancers linked to BRCA gene mutations, representing a pivotal development in the field of oncology (Ledermann et al., 2012). The 2010s marked a pivotal era with the FDA approval of Olaparib in 2014 for ovarian cancer treatment. This milestone was soon followed by the approval of other PARP inhibitors, including Rucaparib, Niraparib, and Talazoparib (Swisher et al., 2017; Mirza et al., 2016; Litton et al., 2018). In recent years, the scope of DDR inhibitors has expanded, integrating these therapies with chemotherapy and immune checkpoint inhibitors to overcome resistance and enhance therapeutic outcomes. Ongoing research continues to explore their potential beyond traditional BRCA-mutant cancers, aiming to broaden their application in cancer therapy.

## 3 Molecular determinants of efficacy in DDR inhibitors

DDR inhibitors are designed to target crucial proteins involved in DNA repair pathways, making them powerful tools for cancer therapy, particularly for cancers heavily reliant on these pathways (Kelley et al., 2014; Teo et al., 2017). Most DDR inhibitors operate on the principle of synthetic lethality, whereby inhibiting a DDR pathway is fetal to cells already deficient in a complementary DNA repair mechanism (e.g., PARP inhibitors in BRCA-mutated cancers) (Yang et al., 2012). These inhibitors capitalize on the genetic instabilities and repair deficiencies common in cancer cells, aiming to block DNA repair and thereby induce cancer cell death (Kelley et al., 2014). The efficacy of DDR inhibitors, such as DNA-PKcs, PARP, ATR, ATM, Chk1/2, and Wee1 inhibitors, depends substantially on their specific molecular targets within the DDR pathways. The activation of these targets is influenced by the types of DNA damage and the genetic context of the cancer cells being treated. Each category of inhibitor interacts differently with its target, highlighting the importance of understanding the underlying molecular and genetic mechanisms to optimize therapeutic outcomes. Below is a closer examination of why some DDR inhibitors are more effective than others, grounded in the underlying molecular biology.

DNA-PKcs Inhibitors: DNA-PKcs is a key component of the NHEJ pathway, responsible for repairing DSBs. DNA-PKcs inhibitors impede the ability of cancer cells to repair these breaks, which is particularly important in rapidly dividing cells. These inhibitors are most effective in tumors with high rates of DSBs and deficient in other repair pathways like HR (Shrivastav et al., 2009).

ATM Inhibitors: ATM is activated by DSBs and plays a role in repair through HR. ATM inhibitors block this process, leading to cell death in tumors that rely on ATM for survival (Durocher and Jackson, 2001). However, developing ATM inhibitors has been challenging due to ATM’s essential role in normal cell DNA repair (Pilié et al., 2019), and their effectiveness is limited in cancers where alternative pathways, like ATR, can compensate for the loss of ATM function.

PARP Inhibitors: PARP inhibitors, such as Olaparib, target PARP1 and PARP2 enzymes involved in the BER pathway, essential for repairing SSBs. In cells deficient in HR, such as those with BRCA1/2 mutations, inhibiting PARP leads to the accumulation of DNA damage and subsequent cell death, a phenomenon known as synthetic lethality (Lord and Ashworth, 2017). The effectiveness of PARP inhibitors highly depends on the presence of HR deficiencies. Some PARP inhibitors, like Talazoparib, exhibit a strong ability to “trap” PARP on DNA, which can lead to greater cytotoxicity but also increased side effects (Murai et al., 2012).

ATR Inhibitors: ATR kinase is activated in response to replication stress and helps stabilize replication forks, preventing their collapse and the formation of DSBs (Lecona and Fernandez-Capetillo, 2018; Karnitz and Zou, 2015). ATR inhibitors are particularly effective in cancers with high levels of replication stress or when used in combination with agents that induce replication stress. However, the effectiveness of these inhibitors can be limited in tumors with intact DDR pathways or in cases where alternative repair mechanisms compensate for ATR inhibition.

Chk1 and Chk2 Inhibitors: Chk1 and Chk2 are checkpoint kinases that regulate cell cycle progression in response to DNA damage (Bartek and Lukas, 2003). Chk1 is particularly critical during the S and G2 phases of the cell cycle, making its inhibition potentially lethal to rapidly dividing cancer cells. Chk1 inhibitors tend to be more effective in cancers where the G1/S checkpoint (controlled by p53) is dysfunctional, forcing the cells to rely heavily on the S/G2 checkpoint for survival (Merry et al., 2010). In contrast, Chk2 has more redundancy and is less commonly targeted alone (Antoni et al., 2007).

Wee1 Inhibitors: Wee1 kinase is a critical regulator of the G2/M checkpoint; inhibition of this kinase propels cells harboring DNA damage into premature mitosis. This premature entry into mitosis leads to mitotic catastrophe, ultimately resulting in cell death (Do et al., 2013; Geenen and Schellens, 2017). Wee1 inhibitors are particularly effective in cancers that depend heavily on the G2/M checkpoint, such as those with p53-deficient. However, their effectiveness can be limited in tumors that can employ alternative mechanisms to regulate the cell cycle or handle mitotic stress.

In summary, the functionality of DDR inhibitors is intricately associated with the specific molecular pathways they target, as well as the genetic and cellular context of the tumors. The efficacy of DDR inhibitors is proportionally related to the extent to which cancer depends on the specific pathway targeted by the treatment. Conversely, tumors equipped with compensatory pathways or those lacking specific vulnerabilities may exhibit reduced responsiveness. This highlights the importance of precision medicine in selecting the most appropriate DDR inhibitor based on the molecular and genetic profile.

## 4 Advances in the applicaiton of treatment based on DDR

### 4.1 Advances in the application of DNA-PKcs inhibitors

DNA-PK is pivotal in NHEJ, a key DNA repair mechanism (Goodwin and Knudsen, 2014; Lieber, 2010). The Ku70/Ku80 complex recognizes and binds to broken DNA ends, recruiting monomeric DNA-PKcs to form an active DNA-PK complex. This complex serves as a scaffold that bridges the DNA ends, facilitating the recruitment and phosphorylation of repair proteins such as Ku70, Ku80, Artemis, XRCC4, XLF, and DNA Ligase IV, which are critical for completing the repair process. Research has shown that tumor cells often upregulated DNA-PKcs expression following radiotherapy or chemotherapy to repair damaged DNA and evade cell death, leading to acquired resistance against these therapies (Goodwin and Knudsen, 2014; Damia, 2020; Hu et al., 2021; Pospisilova et al., 2017). Thus, DNA-PKcs emerges as a promising target for anticancer therapy. Effectively suppressing DNA-PKcs with inhibitors, when used alongside radiotherapy or chemotherapy, can help overcome tumor cell resistance and enhance therapeutic outcomes.

Four DNA-PKcs inhibitors are currently in Phase I/II clinical trials: AZD7648, M9831, M3814, and CC-115. AZD7648 stands out for its high selectivity, showing over 100-fold specificity for DNA-PKcs compared to related kinases such as ATM, ATR, PI3Kα, PI3Kβ, and PI3Kδ (Goldberg et al., 2020). It is currently under evaluation in the Phase I trial for adult soft tissue sarcoma therapy (NCT05116254). Another completed study, NCT03907969, explored AZD7648 as a single agent and combined it with other anticancer therapies for advanced cancers, underscoring its potential for wider oncological use. M9831 is a DNA-PKcs inhibitor known for effectively suppressing NHEJ, thereby impeding the repair of DSBs induced by chemotherapy or radiotherapy (Timme et al., 2018). A clinical trial (NCT02644278) has also been completed investigating M9831 as a monotherapy or combined with PEGylated liposomal doxorubicin. M3814 is a potent DNA-PKcs inhibitor that sensitizes various cancer cell lines to agents inducing DSBs and ionizing radiation (Zenke et al., 2020). Several clinical trials assessing M3814 as a monotherapy or combined with radiotherapy and chemotherapy are ongoing (NCT04555577, NCT04092270). CC-115, a novel dual inhibitor targeting mTOR and DNA-PKcs, demonstrated promise as a well-tolerated and potentially groundbreaking anticancer therapy in a Phase I trial (NCT01353625) (Munster et al., 2019). A subsequent Phase I trial (NCT02833883) combining CC-115 with enzalutamide demonstrated good tolerability in treating metastatic castration-resistant prostate cancer (Zhao et al., 2024). Additionally, preliminary outcomes from a Phase II trial (NCT02977780) focused on innovative glioblastoma therapy revealed that although CC-115 was associated with significant treatment-related toxicity (≥ grade 3) in 58% of patients, it regrettably failed to deliver benefits in terms of progression-free survival (PFS) or overall survival (OS) (Rahman et al., 2023).

Despite significant clinical advancements with DNA-PKcs inhibitors, several challenges remain: 1) Limited Selectivity: Achieving optimal selectivity for DNA-PKcs over closely related kinases, such as PI3K (PI3Kα, β, δ, γ) and other PI3K-related kinases (PIKKs) like ATM and ATR, is challenging due to the high degree of sequence homology. To clarify, while DNA-PK inhibitors have the general trend of limited selectivity in the broader context, AZD7648 is still highlighted as a notable exception, which sets AZD7648 apart from many other inhibitors in this class, exhibiting broader kinase activity profiles and associated off-target effects. 2) Structural Limitations: The considerable molecular weight of DNA-PKcs presents technical difficulties in obtaining its crystal structure. Only the crystal structure of complexes formed by PI3Kγ and DNA-PKcs inhibitors has been elucidated.31 The lack of structural information limits the precise and rational design of highly selective DNA-PKcs inhibitors using computational simulations. 3) Potential Side Effects: Inhibiting DNA-PKcs can adversely affect normal tissues due to its critical role in DDR and repair mechanisms. The impairment of DNA repair in normal tissues can cause toxicity in rapidly dividing tissues, such as the bone marrow and gastrointestinal tract, leading to side effects like myelosuppression and gastrointestinal disturbances. Therefore, the therapeutic window for DNA-PKcs inhibitors must be carefully managed to optimize the anticancer efficacy while minimizing adverse effects on normal tissues. Despite demonstrating excellent anticancer efficacy in animal models (Fok et al., 2019; Gordhandas et al., 2022), standalone DNA-PKcs inhibitors have shown limited clinical efficacy. Future development strategies will likely involve combination therapies to enhance their anticancer effects. Additionally, researchers will focus on developing more selective, efficacious, and less toxic DNA-PKcs inhibitors to further improve therapeutic outcomes.

### 4.2 Advances in the application of PARP inhibitors

BRCA-deficient tumor cells heavily rely on PARP-mediated single-strand DNA repair pathway due to defects in double-strand DNA repair, making PARP a widely utilized anticancer target (Dziadkowiec et al., 2016). PARP inhibitors can function as sensitizers in chemotherapy and radiotherapy by inducing synthetic lethality in DNA damage, thereby augmenting the therapeutic efficacy of these treatments (Kamel et al., 2018). Mechanistically, upon oxidative stress or alkylation damage to DNA, PARP1 activation leads to the recruitment of nucleases such as MRE1 and Exo1 to assist in DNA repair, thereby preserving genome stability. Inhibition of PARP1 disrupts the DNA damage repair pathway, resulting in aberrant apoptosis or cell death.

To date, four PARP1 selective inhibitors have been approved by US FDA for treating malignant tumors. Among these, Talazoparib, a next-generation PARP inhibitor, received approval in 2018 for patients with metastatic or locally advanced breast cancer carrying BRCA mutations based on EMBRACA (NCT01945775) (Hoy, 2018). Another Phase III trial evaluating the combination of Talazoparib and Enzalutamide in men with first-line metastatic castration-resistant prostate cancer has demonstrated a clinically and statistically significant improvement in radiographic progression-free survival (rPFS) compared to treatment with enzalutamide alone. Final OS data and extended safety follow-up are underway, which will provide further insight into the long-term clinical benefits of this treatment regimen (Agarwal et al., 2023). In 2016, US FDA approved Rucaparib as a third-line treatment for female ovarian cancer patients (Shirley, 2019). Additionally, Rucaparib was evaluated in a phase II trial (NCT03413995) as a monotherapy for patients with metastatic hormone-sensitive prostate cancer who have germline mutations in HR repair genes. However, this trial was terminated early due to failing to meet its pre-specified efficacy threshold, leading to discontinuation of enrollment (Markowski et al., 2024). Niraparib received US FDA approval in 2017 for the treatment of primary peritoneal cancer, fallopian tube cancer, or recurrent epithelial ovarian cancer that is resistant to first-line platinum-based chemotherapy (Heo and Duggan, 2018). A Phase III trial (NCT01905592) comparing Niraparib to a physician’s choice of treatment in HER2-negative, germline BRCA mutation-positive breast cancer patients was also terminated early due to insufficient efficacy. Olaparib first obtained FDA approval in 2014 for treating germline BRCA-mutated advanced ovarian cancer after three or more prior lines of chemotherapy (Bochum et al., 2018). In 2017, it was approved for the maintenance treatment of adults with recurrent epithelial ovarian, fallopian tube, or primary peritoneal cancer in a complete or partial response to platinum-based chemotherapy (FDA, 2017). Furthermore, in 2018, Olaparib became the first PARP inhibitor to receive FDA approval for treating germline BRCA-mutated HER2-negative metastatic breast cancer after three or more prior lines of chemotherapy (FDA, 2018). Veliparib remains under investigation and has not yet received FDA approval. It has shown potential in enhancing the effects of several chemotherapeutics and has been included in numerous clinical trials of combination therapies (Table 1).

Combining PARP inhibitors with anti-angiogenic therapy has emerged as a recent research focus. Multi-kinase inhibitors targeting VEGFR, PDGFR, and FGFR can induce hypoxic environments and HR deficiency by inhibiting angiogenesis, thereby augmenting the sensitivity of tumor cells to PARP1/2 inhibitors (Ahn and Bekaii-Saab, 2020; Ivy et al., 2016). Furthermore, combining PARP inhibitors with alkylating agents such as Temozolomide and platinum-based drugs can enhance the “synthetic lethality” effect, leading to more effective tumor cell eradication (Lok et al., 2017). Currently, clinical studies are underway investigating the combination of Olaparib/Talazoparib with Temozolomide for the treatment of gliomas, small cell lung cancer (SCLC), and uterine smooth muscle tumors (Hanna et al., 2020). Additionally, combining PARP inhibitors with PD-1/PD-L1 inhibitors has demonstrated a higher overall response rate (ORR) of 71% in patients with platinum-sensitive recurrent ovarian cancer harboring BRCA mutations (Lampert et al., 2020). Niraparib combined with Pembrolizumab has achieved an ORR of 24% and a disease control rate (DCR) of 67% in treating platinum-resistant recurrent ovarian cancer patients (Konstantinopoulos et al., 2019). In conclusion, PARP inhibitors have exhibited significant therapeutic potential across a diverse range of cancers, both as standalone treatments and in combination with other therapies.

### 4.3 Advances in the application of ATR/ATM inhibitors

ATR and ATM are essential partners in synthetic lethality and cancer therapy. Numerous interactions between ATR and ATM signaling pathways ensure genome stability and cell survival (Burma et al., 2001; Matsuoka et al., 2007; Yang et al., 2004). Upon activation by RPA-coated single-stranded DNA, ATRIP binds directly to RPA, localizing ATR to DNA damage sites. This action triggers the ATR-Chk1 signaling cascade, leading to cell cycle arrest at the G2-M phase, thus providing a temporal window for DNA damage repair. Conversely, ATM responds to DSBs by interacting with MRN complex (MRE11-RAD50-NBS1), generatingγ-H2AX and subsequently phosphorylating and activating Chk2. This activation triggers G1-S checkpoints and delays entry to the S phase, facilitating DNA damage repair.

The synergistic effect of inhibiting both ATR and ATM is based on the critical interdependence of their pathways in managing DNA damage, particularly under conditions of oncogenic replication stress and therapeutic interventions such as radiation or chemotherapy. Inhibition of ATR leads to the accumulation of ssDNA regions due to replication stress, which can cause replication forks to collapse and the formation of DSBs. Normally, ATM would be activated by these DSBs to initiate repair. However, when ATM is also inhibited, the repair of these breaks is severely compromised, leading to a buildup of unrepairable DNA damage. This dual inhibition overwhelms the cancer cell repair mechanisms, significantly enhancing cell death. This strategy provides a strong rationale for the combined use of ATR and ATM inhibitors, particularly in tumors that heavily depend on these pathways due to existing DNA repair deficiencies.

Several ATR/ATM inhibitors have entered clinical trials for cancer treatment. AZD6738 is an effective oral bioavailable ATR inhibitor (Min et al., 2017). In a Phase I trial (NCT02630199), AZD6738 was administered at 240 mg twice daily in combination with paclitaxel, achieving a promising ORR of 22.6%, which increased to 33.3% in the melanoma subgroup. The median progression-free survival (mPFS) was 3.6 months, and the median overall survival (mOS) was 7.4 months, with the most common adverse reactions being neutropenia (68%), anemia (44%), and thrombocytopenia (37%) (Kim et al., 2021). M6620, another specific ATR inhibitor, significantly inhibits pancreatic tumor growth without notable toxicity to normal cells or tissues (Thomas et al., 2018). A Phase I trial (NCT03641547) demonstrated that combining M6620 with radiation therapy is feasible and well-tolerated in esophageal cancer patients. Its use with cisplatin and capecitabine also showed tolerability in advanced cancer cases (Javed et al., 2024). Another Phase I study (NCT02487095) demonstrated that combining M6620 with Topotecan is especially effective in treating platinum-refractory small-cell lung cancer. This condition does not respond well to Topotecan alone.59 BAY-1895344 is a potent, highly selective, orally available ATR inhibitor, demonstrating significant efficacy as a monotherapy in cancer xenograft models with specific DNA damage repair deficiencies (Lücking et al., 2020). A Phase I clinical study (NCT03188965) investigating BAY-1895344 for treating patients with advanced solid tumors and lymphomas has completed recruitment. Initial results from this study, involving 22 patients, indicated that four achieved partial responses. Furthermore, patients exhibiting ATM mutations or loss had a median survival time of 315.5 days. Overall, BAY-1895344 is well-tolerated and shows antitumor activity in cancers with certain DDR defects, including ATM loss (Yap et al., 2021). However, another Phase I clinical study (NCT04267939), testing BAY-1895344 in combination with Niraparib, was terminated as the experimental combination did not provide the anticipated benefits over existing standard therapies. M4344, a potent ATR kinase inhibitor that effectively suppresses Chk1 phosphorylation (Gorecki et al., 2020), encountered challenges in its development. A Phase I study (NCT02278250) revealed that while M4344 was well-tolerated at lower doses, unexpected liver toxicity at higher doses could limit its therapeutic efficacy (Burris et al., 2024). Two additional trials of M4344 (NCT04149145/NCT04655183) were withdrawn for undisclosed reasons. M1774 is a potent ATR inhibitor currently in the recruitment phase for several clinical trials targeting various types of cancer (Table 1).

AZD0156 is an oral ATM inhibitor that efficiently blocks ATM kinase activity, induces apoptosis in malignant tumors, and leads to tumor cell death (Pike et al., 2018). The pharmacokinetics, tolerability, safety, and efficacy of AZD0156 are being evaluated in a Phase I clinical trial, which has completed recruitment (NCT02588105). AZD1390 is an orally active, CNS-penetrating ATM inhibitor distinguished by its exceptional selectivity for ATM, demonstrating potency more than 10,000 times greater than other enzymes in the PIKK family. It is also in the recruitment phase for multiple clinical trials aimed at treating various types of cancer (Table 1). These developments highlight the ongoing efforts to harness the therapeutic potential of ATR/ATM inhibitors in oncology.

Despite these developments, no ATM/ATR inhibitors have been approved for clinical use. The potential for tumors to bypass inhibited ATR/ATM pathways via alternative mechanisms underscores the importance of combining ATM or ATR inhibitors with PARP inhibitors. Cancers with ATM mutations often rely more heavily on ATR for survival and DNA repair (Armstrong et al., 2019; Cheng et al., 2022). This dependency makes ATR an appealing target because inhibiting ATR in these contexts can specifically sensitize cancer cells to treatment without similarly affecting healthy cells. This combination could provide a synergistic effect, enhancing antitumor outcomes through synthetic lethality. ATR is one of the most promising synthetic lethality targets, holding significant potential for treating cancers with ATM mutations or loss. These efforts continue to demonstrate the significant potential of ATR/ATM inhibitors in oncology.

### 4.4 Advances in the application of Chk1/Wee1 inhibitors

Overexpression of Chk1 and Wee1 has been observed in various cancers, including ovarian and breast cancer (Cleary et al., 2020; Ghelli Luserna Di Rorà et al., 2019). Inhibiting Wee1 or Chk1 causes tumor cells with DNA damage to enter mitosis prematurely, leading to apoptosis or cell death. Mechanistically, single-stranded DNA damage activates ATR, which phosphorylates and activates Chk1. This activation subsequently phosphorylates cdc25C and Wee1, leading to the activation of Wee1 and inhibition of cdc25C. Wee1 further phosphorylates the CDK1-Cyclin B complex, rendering it inactive and causing G2 arrest to allow for DNA repair (Du et al., 2020; Matheson et al., 2016). As a critical protein kinase, Wee1 effectively inhibits CDK2 and CDK1 to activate the G2/M checkpoint, inducing G2/M arrest to allow for DNA repair. Inhibiting Wee1 prevents the G2 checkpoint initiation, allowing cells to enter mitosis with incorrect DNA content, leading to a loss of genomic integrity and cell death. Cancer cells with dysregulated G1/S cell cycle checkpoints heavily rely on the G2/M checkpoint to prevent excessive DNA damage accumulation. Therefore, based on synthetic lethality, inhibiting Wee1 can block the G2/M checkpoint to treat p53-deficient tumor cells, as p53 plays a critical role in the G1 checkpoint. Current research primarily focuses on combining Wee1 inhibitors with other therapeutic agents that induce DNA damage, including PARP inhibitors, chemotherapy, or radiotherapy for patients carrying TP53 mutations. Chk1 is a highly conserved serine/threonine kinase that is involved in multiple signal transduction pathways activated by DNA damage events (Dent, 2019; Zhang and Hunter, 2014). Inhibiting Chk1 can disrupt the G2 checkpoint initiation, impair DNA repair and promote tumor cell apoptosis (Carrassa and Damia, 2011; Rundle et al., 2017).

LY-2606368 is a potent Chk1 kinase inhibitor with an IC50 of less than 1 nM for Chk1 and less than 8 nM for Chk2 (Heidler et al., 2020). It has shown promise in a Phase II clinical trial (NCT03414047), demonstrating durability as a single agent in certain patients with recurrent ovarian cancer (Konstantinopoulos et al., 2022). Another study comparing the activity and off-target effects of CHK1 inhibitors MK-8776, SRA737, and LY2606368 demonstrates that LY2606368 is the most selective CHK1 inhibitor (Ditano and Eastman, 2021). This finding supports the potential for further clinical development of LY2606368. Another Chk1 kinase inhibitor, GDC-0575, is known to enhance the sensitivity of cancer cells to chemotherapy-induced DNA damage (Li et al., 2021). Although GDC-0575 can be safely administered alone or in combination with gemcitabine, its antitumor efficacy was limited, achieving only a 15% PR rate among 102 patients treated with the combination in a Phase I study (NCT01564251) (Italiano et al., 2018). SRA737, a different orally active Chk1 inhibitor, was well-tolerated in Phase I/II trials (NCT02797964) focusing on solid tumors. However, it lacked sufficient efficacy as a monotherapy. Future studies should explore its use in combination therapies (Kristeleit et al., 2023). MK-8776 also exhibited strong and selective inhibition of Chk1 and was well-tolerated either alone or in combination with gemcitabine in a Phase I trial with advanced solid tumors (Daud et al., 2015). However, a randomized Phase II trial exploring the efficacy of cytosine arabinoside with and without MK-8776 in relapsed and refractory acute myeloid leukemia found that while MK-8776 significantly increased DNA damage in leukemia cells, as indicated by elevated γ-H2AX levels, it did not lead to notable improvements in treatment responses or survival outcomes compared to the control group (Webster et al., 2017).

AZD1775, the pioneering Wee1 inhibitor to enter Phase I trial (NCT02610075), has demonstrated tolerability and efficacy in reducing tumor size in patients with advanced solid tumors. The inhibitor specifically targets the G2/M checkpoint, increasing the vulnerability of p53-deficient tumors to DNA damage induced by radiotherapy or chemotherapy. Zn-C3, another orally active, potent, and selective Wee1 inhibitor, is currently being evaluated in multiple clinical trials for a range of cancers, including ovarian cancer, fallopian tube cancer, uterine carcinoma, peritoneal cancer, acute myeloid leukemia, colorectal cancer, breast cancer, osteosarcoma and other solid tumors (Table 1). Additional novel Wee1 inhibitors such as Debio 0123, SY-4835, and IMP7068 are now entering Phase I clinical trials (NCT03968653, NCT05291182, NCT04768868), with hopes for positive outcomes.

## 5 Lessons from unsuccessful DDR inhibitor trials in oncology

The failure of clinical trials involving DDR inhibitors, such as those targeting PARP, ATR, and other key proteins, has provided critical insights for the future of tumor drug development. Several common factors contributing to these failures offer valuable lessons: 1) Biological Complexity and Tumor Heterogeneity: DDR pathways are inherently complex and often exhibit significant redundancy. Tumors can adapt by activating alternative survival pathways, which reduces the efficacy of DDR inhibitors. To overcome this challenge, a deeper understanding of tumor biology and heterogeneity is essential. Conducting biomarker-driven trials and developing companion diagnostics are crucial for identifying patients who are most likely to benefit from specific DDR inhibitors. Additionally, refining drug designs to boost specificity and minimize off-target effects could significantly improve the therapeutic potential of these inhibitors. 2) Inadequate Preclinical Models: Promising results from preclinical models often fail to translate into clinical success, largely because these models inadequately represent human tumors. To improve the predictive accuracy of preclinical studies, it is essential to adopt more representative models, such as patient-derived xenografts and organoids, which more closely mimic the biological complexities of human tumors. 3) Toxicity and Side Effects: DDR inhibitors can cause significant toxicity, particularly when combined with other treatments, such as chemotherapy or radiation. Optimal patient selection, precise dosing strategies, and comprehensive phase I studies to delineate toxicity profiles are critical steps in mitigating these risks. It is also important to base combination therapies on strong biological evidence supported by preclinical data to manage and prevent potential toxicities and interactions. 4) Drug Resistance: Resistance to DDR inhibitors can arise through various mechanisms, including mutations in the target enzymes or alterations in drug metabolism. Combination therapies that target multiple pathways simultaneously may help overcome resistance. Continuous monitoring of resistance mechanisms during clinical trials can provide essential feedback for adjusting treatment protocols. In summary, analyzing failed DDR inhibitor trials is crucial for gaining strategic insights that can improve trial designs, refine patient selection criteria, and optimize therapeutic strategies. These lessons are vital for increasing the probability of success in future oncological drug development endeavors.

## 6 Challenges and prospects

Recent advancements in DDR inhibitors for cancer therapy have ushered in a landscape filled with both significant challenges and immense prospects. Key challenges include managing severe side effects such as leukopenia and myelotoxicity, especially when these inhibitors are used alongside chemotherapy. There is also a critical need to enhance the selectivity and specificity of these therapies to minimize their impact on healthy cells, expand their therapeutic windows, and identify more genetic biomarkers that can accurately predict patient responses to treatments. On the promising side, ongoing research dedicated to refining molecular designs is leading to the development of more effective and less toxic drugs. These innovative inhibitors are precisely engineered to target specific DDR deficiencies, potentially expanding the range of treatable cancers. Furthermore, optimizing combination therapies aims to reduce the required dosages and overall cumulative toxicity, thereby transforming treatment outcomes. Looking forward, the trajectory of DDR inhibitors involves surmounting these existing limitations through groundbreaking drug design and advanced clinical strategies, potentially enabling their widespread implementation in cancer therapy protocols and significantly improving survival rates. These developments are poised to convert non-responders into responders and elevate existing responders to “super-responder” status, revolutionizing the cancer treatment landscape with more personalized, effective, and safer therapeutic options.

## 7 Conclusion

Significant advancements in targeted DNA repair inhibition for cancer therapy have been made, marked by the development of highly selective and efficacious DDR inhibitors. These therapies leverage synthetic lethality to tailor treatment for cancer patients with specific DDR deficiencies, achieving precision and personalized treatment outcomes. By inhibiting DDR, these therapies enhance the effectiveness of related drugs and overcome treatment resistance by preventing cancer cells from repairing DNA damage. However, the potential of DDR inhibitors comes with challenges, particularly when combined with chemotherapy, which can lead to side effects such as leukopenia, gastrointestinal toxicity, and myelotoxicity. Future research on DDR inhibitors will focus on improving their selectivity and specificity to reduce toxicity and minimize the dosage required for combination therapy. Another challenge is broadening their therapeutic windows and identifying additional genetic biomarkers sensitive to DDR inhibition. PARP inhibitors are the only FDA-approved DDR inhibitor due to their wide therapeutic windows. Further efforts will aim to discover more biomarkers with therapeutic relevance, potentially expanding the patient population that benefits from DDR inhibitors.

## Abbreviations

DCR, disease control rate; DDR, DNA damage response; DSBs, double-strand breaks; HR, homologous recombination; MMR, mismatch repair; mOS, median overall survival; mPFS, median progression-free survival; NER, nucleotide excision repair; NHEJ, non-homologous end joining; ORR, overall response rate; OS, overall survival; PFS, progression-free survival; PIKKs, PI3K-related kinases; rPFS, radiographic progression-free survival; SCLC, small cell lung cancer; SSBs, single-strand breaks; SCLC, small cell lung cancer.
